# Reprogramming the Circadian Dynamics of Epileptic Genes in Mouse Temporal Lobe Epilepsy

**DOI:** 10.3390/ijms24076400

**Published:** 2023-03-29

**Authors:** Sha Sun, Han Wang

**Affiliations:** 1 Center for Circadian Clocks, Soochow University, Suzhou 215123, China; sunsha@suda.edu.cn; 2 School of Biology & Basic Medical Sciences, Suzhou Medical College, Soochow University, Suzhou 215123, China

**Keywords:** temporal lobe epilepsy, epileptic genes, rhythmicity, circadian clocks, chronotherapy

## Abstract

Temporal lobe epilepsy (TLE) is a common and severe epilepsy displaying rhythmicity in humans and animals. However, how the circadian clock contributes to TLE remains elusive. A recent circadian analysis of the ventral hippocampal transcriptome of pilocarpine-induced TLE mice revealed as many as 1650 rhythmically expressed transcripts. Here, a comparison of the mouse ventral hippocampal transcriptome with the human epilepsy-related gene set identified 315 possible mouse epilepsy-related genes. Rhythmicity analysis classified them into arrhythmicity, loss-of-rhythmicity, gain-of-rhythmicity, and rhythmicity-maintaining groups. KEGG and GO analyses of these mouse epilepsy genes suggest their involvement in circadian entrainment. In TLE mice, *Htr1d*, *Drd2*, and *Chrna3* lose rhythmicity, but *P2rx7* gains rhythmicity; the up-regulation of *Htr1d* and *Drd2* and down-regulation of *Chrna3* inhibit adenylate cyclase (AC), and up-regulation of *Htr1d*, *Drd2*, and *P2rx7* activates protein kinase C (PKC). Together, these results suggest that epilepsy can disrupt the circadian dynamics of the epileptic genes, shed light on possible TLE pathogenesis, and provide potential targets for TLE diagnosis and chronotherapy.

## 1. Introduction

Temporal lobe epilepsy (TLE) is a common severe neurologic disorder characteristic of recurrent seizures [1]. The potential role of the circadian clock in seizures has been observed in humans and animals [2,3]; for instance, neocortical temporal lobe epilepsy (NTLE) peaks in the afternoon in humans [4] and displays a diurnal pattern in rodent models [5]. The rhythmic expression of canonical circadian clock genes is disrupted in human epileptic tissues [6], and mouse mutants of core circadian clock genes exhibit increased epileptic susceptibility [6]. However, whether and how the rhythmicity of epilepsy-related genes is altered in epilepsy remains unclear.

A recent circadian analysis of the ventral hippocampal transcriptome of the pilocarpine-induced TLE mice revealed abnormal expression patterns of genes involved in aerobic glycolysis and oxidative phosphorylation in TLE mice [3], shedding light on the altered circadian transcription dynamics in the ventral hippocampal transcriptome of TLE mice. Moreover, this study also provides an opportunity to investigate the altered rhythmicity of mouse epilepsy-related genes.

Here, we interrogated the ventral hippocampal transcriptome of mice with 661 human epilepsy-related genes we recently compiled [7] and identified 315 possible mouse epilepsy-related genes. Rhythmicity analysis classified them into four groups. KEGG and GSEA analyses show that the circadian entrainment pathway is likely repressed in TLE mice. We find that *Htr1d*, *Drd2*, and *Chrna3* involved in the neuroactive ligand-receptor interaction pathway lose rhythmicity, but *P2rx7* gains rhythmicity, and their up-regulation or down-regulation results in the down-regulation of adenylate cyclase (AC) and up-regulation of protein kinase C (PKC) in TLE mice, providing potential targets for TLE diagnosis and chronotherapy.

## 2. Results

### 2.1. Epilepsy-Related Genes in the Ventral Hippocampal Transcriptome of Mice

**Figure 1:**
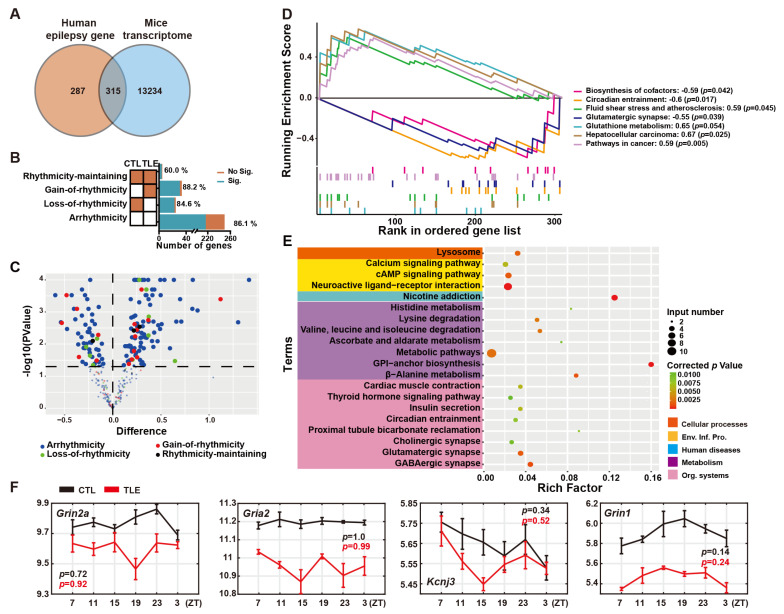
Analysis of mouse epileptic genes. (**A**) Venn diagram of 315 epilepsy-related genes in the ventral hippocampus of mouse temporal lobe epilepsy (TLE). (**B**) Classification of 315 possible mouse epilepsy-related genes into 251 arrhythmic genes, 25 loss-of-rhythmicity genes, 34 gain-of-rhythmicity genes, and five rhythmicity-maintaining genes. Shown are percentages of significantly differentially expressed genes between TLE and CTL groups (blue) and not significantly differentially expressed genes (orange). (**C**) Volcano plot of up-regulated and down-regulated genes in arrhythmicity, loss-of-rhythmicity, gain-of-rhythmicity, and rhythmicity-maintaining groups at the seizure peak time (ZT7) of mouse TLE. (**D**) Gene set enrichment analysis (GSEA) of significantly altered KEGG pathways in 315 epilepsy-related genes at ZT7. The rank of the gene set of each pathway is listed on the right side of the plot. Four pathways show positive enrichment scores, and three show negative enrichment scores. (**E**) Top 20 KEGG enrichment pathways of 79 mouse epilepsy driver genes. Circles represent the gene numbers in specific pathways and colored intensities the corrected *p* values. The first-level categories of KEGG pathways are marked in different colors. (**F**) Expression of representative arrhythmic genes involved in the circadian entrainment and glutamatergic synapse pathways simultaneously, including *Grin2a* (Glutamate ionotropic receptor NMDA type subunit 2A), *Gria2* (Glutamate ionotropic receptor AMPA type subunit 2), *Kcnj3* (Potassium inwardly rectifying channel subfamily J member 3), and *Grin1* (Glutamate ionotropic receptor NMDA type subunit 1). The black curve represents gene expression values from the CTL mice, and the red curve is for those in TLE mice.

In this study, we interrogated the ventral hippocampal transcriptome of mice [3] with 661 human epilepsy-related genes, recently compiled from two widely accepted public disease databases [7], and identified 315 possible mouse epilepsy-related genes (Figure 1A), including 79 causative driver genes, 101 passenger genes, and 135 undetermined genes (Tables S1 and S2). Rhythmicity analysis further classified these mouse epilepsy-related genes into 251 arrhythmic genes, 25 loss-of-rhythmicity genes, 34 gain-of-rhythmicity genes, and five rhythmicity-maintaining genes (Figure 1B, Table S3). High percentages of significantly differentially expressed genes showed the altered expression of most epilepsy-related genes in TLE mice, likely contributing to the rhythmic seizures (Table S4). Up-regulated and down-regulated genes in these four groups around the peaking time of seizures (ZT7) [3] are shown in the Volcano plot (Figure 1C). We observed seven significantly altered KEGG pathways at ZT7 in TLE mice, including four up-regulated pathways and three down-regulated pathways (Figure 1D, Table S5), as determined by gene set enrichment analysis (GSEA). Epilepsy-related genes involved in the circadian entrainment and glutamatergic synapse pathways are down-regulated in TLE mice (Figure 1D), as shown by the expression of *Grin2a* (Glutamate ionotropic receptor NMDA type subunit 2A), *Gria2* (Glutamate ionotropic receptor AMPA type subunit 2), *Kcnj3* (Potassium inwardly rectifying channel subfamily J member 3), and *Grin1* (Glutamate ionotropic receptor NMDA type subunit 1), in TLE and control (CTL) mice (Figure 1F). In addition, numerous epilepsy driver genes involved in the neuroactive ligand-receptor interaction and metabolic pathways are also enriched in TLE mice, as shown by KEGG analysis (Figure 1E, Table S6), implicating altered neuroactive signaling and disrupted metabolism in TLE mice.

### 2.2. Arrhythmic Epilepsy-Related Genes in TLE Mice

The expression of 251 arrhythmic epilepsy-related genes in TLE and CTL mice is shown in the heatmap (Figure S1A). KEGG analysis showed that arrhythmic epilepsy-related genes involved in circadian entrainment, neuroactive ligand-receptor interaction, the cAMP signaling pathway, and the serotonergic synapse pathway are enriched in TLE mice (Figure S1B, Table S7). In particular, four arrhythmic epilepsy-related genes involved in the cAMP signaling and serotonergic synapse pathways, including *Bdnf* (Brain-derived neurotrophic factor), *Gli3* (GLI-Kruppel family member GLI3), *Htr2a* (5-Hydroxytryptamine receptor 2A), and *Gabrb1* (GABA receptor A subunit β1), are up-regulated in TLE mice (Figure S1C). As protein kinase A (PKA) is known to repress *Gli3* [8], up-regulation of *Gli3* implicates the down-regulation of PKA. *Htr2a* activates Ca^2+^ transporters in postsynaptic cells and interacts with Gq to activate Protein kinase C (PKC), indirectly reducing adenylate cyclase (AC) production [9], while phosphorylation of *Gabrb1*, facilitated by the cAMP pathway, is up-regulated in TLE mice [10]. Hence, it appears that the cAMP pathway is disrupted in TLE mice. Further, KEGG analysis also showed that some of these arrhythmic epilepsy-related genes are likely involved in carcinogenesis, autoimmune thyroid disease, and type I diabetes mellitus (Figure S1B).

### 2.3. Loss-Of-Rhythmicity Epileptic Genes in TLE Mice

**Figure 2:**
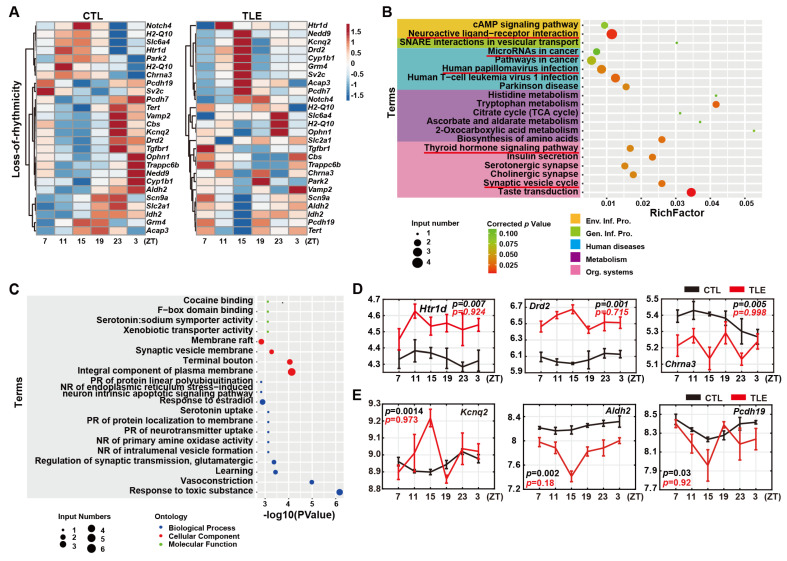
Analysis of 25 loss-of-rhythmicity genes. (**A**) Heatmaps of 25 loss-of-rhythmicity genes in CTL (**left**) and TLE (**right**) mice. (**B**) Top 20 KEGG enrichment pathways of loss-of-rhythmicity genes in TLE mice. Circles represent the gene numbers in specific pathways and colored intensities the corrected *p* values. The first-level categories of KEGG pathways are marked in different colors. The same pathways that also turn up in KEGG analysis of gain-of-rhythmicity genes are underlined. (**C**) Top 20 GO enrichment terms of loss-of-rhythmicity genes. NR: negative regulation, PR: positive regulation. (**D**) Expression of three representative loss-of-rhythmicity genes *Htr1d* (5-hydroxytryptamine receptor 1D), *Drd2* (dopamine receptor D2), and *Chrna3* (cholinergic receptor nicotinic alpha 3 subunit) involved in the neuroactive ligand-receptor interaction pathway. (**E**) Expression of three driver genes in the loss-of-rhythmicity group, including *Kcnq2* (Potassium voltage-gated channel subfamily Q member 2), *Pcdh19* (Protocadherin 19), and *Aldh2* (Aldehyde dehydrogenase 2 family member). *p* value of meta2d of each gene was calculated with MetaCycle. The black curve represents gene expression values from the CTL mice, and the red curve is for those in TLE mice.

The 25 epileptic genes clearly lose rhythmicity in TLE mice, as shown by the heatmap (Figure 2A) and MetaCycle analysis (Table S3). KEGG analysis shows that several epileptic genes involved in the neuroactive ligand-receptor interaction pathway are enriched in TLE mice (Figure 2B, Table S8), while GO analysis shows that other epileptic genes involved in the neurotransmitter uptake process are altered in TLE mice (Figure 2C, Table S9). In particular, two genes, *Htr1d* (5-Hydroxytryptamine receptor 1D) and *Drd2* (Dopamine receptor D2), lose rhythmicity but are up-regulated in TLE mice (Figure 2D). DRD2, as a dopamine transporter, transports dopamine into the presynaptic terminal and postsynaptic cells, inhibiting AC activity through interacting with inhibitory G-proteins (Gi) [11] and activating the PKC pathway via the calcium signaling pathway [12]. Similarly, HTR1D also inhibits AC activity and activates the PKC pathway. On the other hand, *Chrna3* (Cholinergic receptor nicotinic alpha 3 subunit) also loses rhythmicity but is down-regulated in TLE mice (Figure 2D). Nicotinic acetylcholine receptor (nAChR) encoded by *Chrna3* activates AC by binding to acetylcholine and then transporting sodium and calcium into the cells [13]. In addition, three driver genes *Kcnq2* (Potassium voltage-gated channel subfamily Q member 2), *Pcdh19* (Protocadherin 19), and *Aldh2* (Aldehyde dehydrogenase 2 family member) lose rhythmicity with altered expression patterns in TLE mice (Figure 2E).

### 2.4. Gain-Of-Rhythmicity Epileptic Genes in TLE Mice

**Figure 3:**
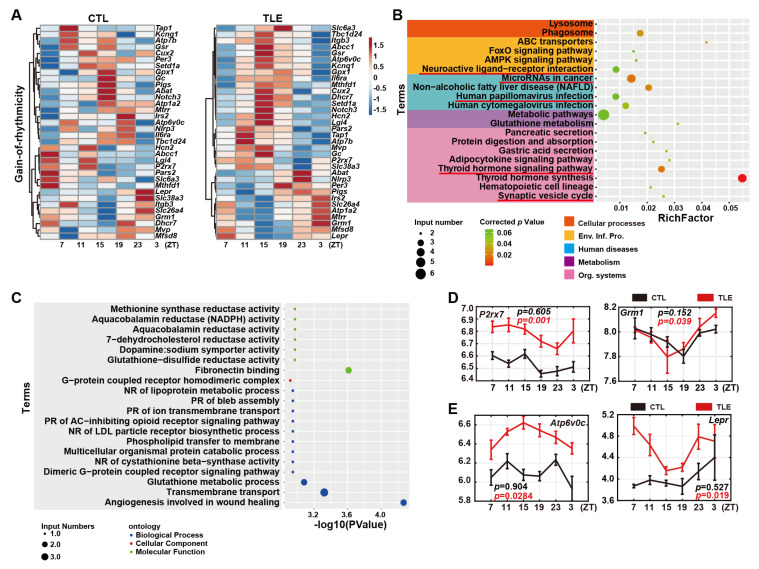
Analysis of 34 gain-of-rhythmicity genes. (**A**) Heatmaps of 34 gain-of-rhythmicity genes in CTL (**left**) and TLE (**right**) mice. (**B**) Top 20 KEGG enrichment pathways of gain-of-rhythmicity genes in TLE mice. The same pathways that also occur in KEGG analysis of loss-of-rhythmicity genes are underlined. (**C**) Top 20 GO enrichment terms of gain-of-rhythmicity genes. (**D**) Expression of two representative gain-of-rhythmicity genes *P2rx7* (purinergic receptor P2X 7) and *Grm1* (glutamate metabotropic receptor 1) involved in the neuroactive ligand-receptor interaction pathway. (**E**) Expression of two driver genes in the gain-of-rhythmicity group, including *Atp6v0c* (ATPase H^+^ transporting V0 subunit c) and *Lepr* (leptin receptor). The black curve represents gene expression values from the CTL mice, and the red curve is for those in TLE mice.

The 34 epileptic genes clearly gain rhythmicity in TLE mice, as shown by the heatmap (Figure 3A) and MetaCycle analysis (Table S3). Intriguingly, KEGG analysis of these 34 gain-of-rhythmicity genes shows the five enriched pathways, such as synaptic vesicle cycle and neuroactive ligand-receptor interaction pathways (Figure 3B, Table S10), which also turn up from KEGG analysis of the 25 loss-of-rhythmicity genes (Figure 2B). Hence, in the same pathways, some epileptic genes lose rhythmicity, while the other epileptic genes gain rhythmicity. For instance, in the neuroactive ligand-receptor interaction pathway, some epileptic genes lose rhythmicity (Figure 3B), and the other epileptic genes gain rhythmicity (Figure 2B). In particular, two genes involved in the neuroactive ligand-receptor interaction pathway, *P2rx7* (purinergic receptor P2X 7) and *Grm1* (glutamate metabotropic receptor 1), gain rhythmicity. P2RX7 transports the calcium into the cytoplasm to up-regulate PKC [14]. GRM1 regulates the calcium levels of the postsynaptic cytosol as a G protein-coupled neurotransmitter receptor, whose mutations mainly result in schizophrenia and bipolar disorder [15], and has been regarded as a potential novel drug target for refractory epilepsy therapy [16]. Further, GO analysis shows that epileptic genes involved in numerous reductase activities are enriched in TLE mice (Figure 3C, Table S11). In addition, two epileptic driver genes, *Atp6v0c* (ATPase H^+^ transporting V0 subunit c) and *Lepr* (leptin receptor) (Table S2), gain rhythmicity and are up-regulated in TLE mice (Figure 3E).

### 2.5. Rhythmicity-Maintaining Epileptic Genes in TLE and CTL Mice

**Figure 4:**
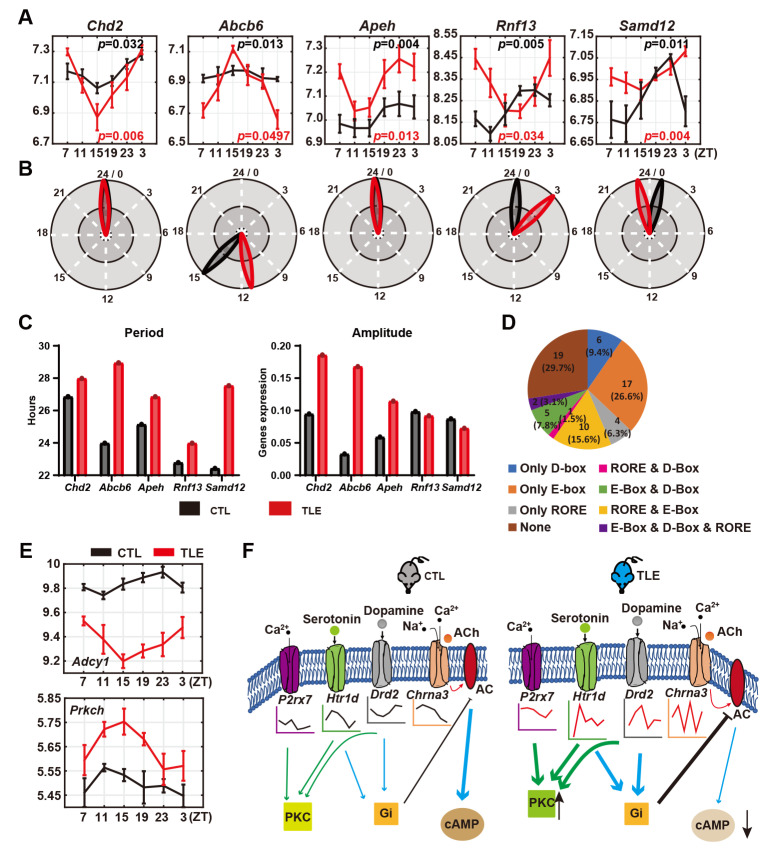
Analyses of five rhythmicity-maintaining epilepsy genes and circadian clock-regulated motifs in the promoters of rhythmically expressed epilepsy genes and the model for the altered cAMP signaling pathway in TLE mice. (**A**) Expression of five representative rhythmicity-maintaining genes *Chd2* (Chromodomain helicase DNA binding protein 2), *Abcb6* (ATP-binding cassette subfamily B member 6), *Apeh* (Acylaminoacyl-peptide hydrolase), *Rnf13* (Ring finger protein 13), and *Samd12* (Sterile alpha motif domain containing 12). (**B**,**C**) Altered phases (**B**), periods, and amplitudes (**C**) of five representative genes in WT and TLE mice. (**D**) Numbers of the epilepsy genes containing E-Box, D-Box, or RORE or their combinations in the 5′ 5000-bp promoter regions. (**E**) Expression of *Adcy1* (Adenylate cyclase 1) and *Prkch* (Protein kinase C, eta type) in CTL and TLE mice. The black curve represents gene expression values from the CTL mice, and the red curve is for those in TLE mice. (**F**) A model for the altered cAMP signaling pathway in TLE mice.

Only five epileptic genes maintain rhythmicity in TLE and CTL mice (Figure 4A, Table S12). Even though the rhythmicity of these five epileptic genes is held in both TLE and CTL mice, their phases, periods, and/or amplitudes are altered in TLE mice. For instance, *Abcb6* (ATP-binding cassette subfamily B member 6), *Rnf13* (ring finger protein 13), and *Samd12* (Sterile alpha motif domain containing 12) are statistically rhythmically expressed in both TLE and CTL mice; however, *Samd12* and *Abcb6* advance approximately 2–4 h, but *Rnf13* delays approximately 3 h in TLE mice (Figure 4B). Even though the phases of *Chd2* (Chromodomain helicase DNA binding protein 2) and *Apeh* (Acylaminoacyl-peptide hydrolase) are not markedly altered, both genes display lengthened periods with elevated amplitudes in TLE mice (Figure 4C). Despite maintaining rhythmicity, altered phases, periods, or amplitudes of these epileptic genes likely exert their effects on the pathogenesis of the rhythmic TLE.

### 2.6. Possible Circadian Regulation of Rhythmically Expressed Epileptic Genes in Mice

To investigate the possible regulatory mechanism underpinning the rhythmic expression of these epileptic genes, we conducted a sequence search for circadian clock-regulated motifs in the 5′ 5000-bp promoter regions of these 64 rhythmically expressed epilepsy genes (Table S13). We found that approximately 70.3% (45) of them harbor either an E-Box, a D-Box, or a RORE or their combinations in the proximal promoter regions (Figure 4D), implicating that the circadian clock may regulate them. Experiments will be needed to verify the circadian regulation of these epileptic genes in the future.

## 3. Discussion

In this study, through interrogating the mouse ventral hippocampal transcriptome [3] with the human epilepsy-related genes [7], we identified 315 possible mouse epilepsy-related genes, including 79 driver genes, 101 passenger genes, and 135 undetermined genes (Tables S1 and S2). GSEA of these 315 epilepsy-related genes showed that the circadian entrainment pathway is down-regulated in TLE mice (Figure 1D), implicating the altered rhythmicity in TLE mice. In particular, the NMDA receptor involved in the circadian entrainment pathway is down-regulated in TLE mice (Figure 1F).

We further conducted a rhythmicity analysis of these mouse epilepsy-related genes with MetaCycle and divided them into arrhythmicity, loss-of-rhythmicity, gain-of-rhythmicity, and rhythmicity-maintaining groups (Figure 1B). An understanding of the alterations of rhythmically expressed epileptic driver genes would help unravel the molecular genetic mechanisms underlying rhythmic epilepsies/seizures and develop effective antiepilepsy drugs and epilepsy chronotherapy [7]. Protocadherin 19 (*PCDH19*), a human epilepsy driver gene, is expressed primarily in the human brain [17], as its mutations have been found in early-onset female-restricted seizures and cognitive disabilities [18,19]. We observed that mouse *Pcdh19* loses rhythmicity and is down-regulated in TLE mice (Figure 2E). ATPase H^+^ transporting V0 subunit c (*ATPV0C*), encoding c-subunit of the vacuolar ATPase, is another human epilepsy driver gene, as it has been reported to cause intellectual impairment and epilepsy [20]. Mouse *Atpv0c* gains rhythmicity but is up-regulated in TLE mice (Figure 3E). In addition, numerous epileptic genes are also likely involved in cancers, autoimmune diseases, and metabolic disorders (Figure S1B), suggesting the co-morbid nature of epilepsies [21].

Approximately 80% (251) of 315 possible mouse epilepsy-related genes are arrhythmic (Table S1), while the remaining 20% (64) genes are either rhythmic in CTL mice but lose rhythmicity in TLE mice (Figure 2A) or are arrhythmic in CTL mice but gain rhythmicity in TLE mice (Figure 3A) or maintain rhythmicity in both CTL and TLE mice (Figure 4A). The KEGG analysis of both the 34 gain-of-rhythmicity genes and the 25 loss-of-rhythmicity genes revealed the five enriched pathways, such as synaptic vesicle cycle and neuroactive ligand-receptor interaction pathways (Figure 2B and Figure 3B), implicating that some epileptic genes lose rhythmicity, but the other epileptic genes gain rhythmicity in the same pathways (Figure 2B and Figure 3B). When a rhythmically expressed gene loses rhythmicity, it implies that its regulated/affected activity or process becomes arrhythmic. In contrast, when an arrhythmic gene gains rhythmicity, it implies that its regulated/affected activity or process becomes rhythmic. Thus, it appears that epilepsy can alter or reprogram the circadian dynamics, making some rhythmic activities or processes lose rhythmicity and also other non-rhythmic activities or processes become rhythmic. Even for those epileptic genes maintaining rhythmicity in TLE and CTL mice, their phases, periods, or amplitudes are disrupted in TLE mice (Figure 4A–C), likely contributing to rhythmic epileptogenesis. Hence, rhythmicity alterations of epileptic genes in TLE mice appear complicated, and their pathogenesis implications should be investigated in the future.

Intriguingly, PKC contributes to the circadian entrainment regulation [22], and the AC-cAMP pathway displays a rhythmicity [23]. Further, PKC is also involved in epilepsy, as evidenced that PKC is markedly increased in the hippocampus of epileptic rats [24], and an AED treatment significantly up-regulates AC, cAMP, and cAMP-response element binding protein (CREB) in the hippocampus of epileptic rats, compared with the untreated epilepsy group [25]. We observed the down-regulation of *Adcy1* (Adenylate cyclase 1) and up-regulation of *Prkch* (Protein kinase C eta type) in TLE mice, implicating the reduced AC levels and enhanced PKC levels in TLE mice (Figure 4E), respectively. As shown in the proposed model (Figure 4F), in wild-type control mice, *5-HT1d* and *Drd2* are rhythmically expressed, and their encoded proteins inhibit the cAMP synthesis and activate PKC synthesis by transporting serotonin and dopamine into the cytoplasm and interacting with Gi, while the rhythmically expressed *Chrna3* encoded nAChR increases the cAMP level by transporting sodium and calcium into the cytoplasm to activate AC. The purinergic receptor *P2rx7*, activated by neurotransmitters, transports calcium into the cytoplasm to increase PKC synthesis. In contrast, in TLE mice, *5-HT1d*, *Drd2*, and *Chrna3* all lose rhythmicity; up-regulation of *5-HT1d* and *Drd2* leads to stronger repression of AC synthesis and activation of PKC synthesis, while down-regulation of *Chrna3* results in fewer activities of nAChR and reduced AC levels. P2rx7 gains rhythmicity in TLE mice, which leads to up-regulated PKC in TLE mice. Collectively, down-regulated cAMP signaling and up-regulated PKC signaling are observed in TLE mice.

However, our study has several limitations. First, the original study analyzed the pilocarpine-induced TLE and control mice with only six time points for one day [3]. Even though the pilocarpine-induced TLE mice can recapitulate clinic syndromes of severe seizures, cognitive impairments, and loss of GABAergic interneurons in the dentate gyrus [26], as a drug-induced animal, these phenotypes and the expression levels of underlying genes vary to a certain degree due to drug dosages, animal ages, and treatment duration [26]. The time-course microarray analysis with the six time points for 24 h often led to an underestimated number of rhythmically expressed genes and a less robust rhythmicity analysis [27]. Second, the 661 human epilepsy-related genes will need to be revised and updated with new experimental verification [7], and therefore their mouse orthologs will need to be updated. Third, as we focused on bioinformatic analysis of this validated and confirmed microarray data set [3], we did not perform independent qRT-PCR experiments to verify some of our analytic results, which would strengthen them. Finally, our comparative analysis provided all mouse orthologs of human epileptic genes in the mouse ventral hippocampus. Thus, some of these 315 mouse epileptic genes may not be directly related to TLE and, therefore, not directly responsive to pilocarpine treatment, as shown in (Figure 1B).

In summary, our bioinformatic analysis of the time-course TLE mouse data suggests that the altered rhythmicity of epilepsy genes underpins the possible TLE pathogenesis, highlighting the circadian role in epilepsy and providing potential targets for developing chronomodulated epileptic therapeutics [7].

## 4. Materials and Methods

The ventral hippocampal transcriptome data of wild-type control (aat5979_Table_S1) and pilocarpine-induced temporal lobe epilepsy (aat5979_Table_S3) mice used in this study were generated by microarray analysis as previously reported [3], which were deposited in CircadiOmics (http://circadiomics.igb.uci.edu/datasets (accessed on 24 May 2021)) (GEO Accession GSE54652). Briefly, the ventral parts of the hippocampus of wild-type control (CTL) and pilocarpine-induced temporal lobe epilepsy (TLE) mice were collected at six time points each with four replicates in a 4 h interval for a single 24 h period, and total RNAs extracted from these samples were subject to transcriptome analysis using Affymetrix GeneChip Mouse Gene 2.1 ST arrays (no. 902120) [3]. The mouse orthologs of human epilepsy-related genes were determined by comparing the mouse ventral hippocampal transcriptome with the human epilepsy-related gene set [7] with R program Homologene (https://www.rdocumentation.org/packages/homologene (accessed on 24 May 2021)). The KEGG enrichment analysis was conducted with the KOBAS database (Mouse gene symbol is the input type, and *p*-value < 0.05 is a filter, http://kobas.cbi.pku.edu.cn/kobas3/ (accessed on 8 August 2022)) [28], and GO enrichment analysis was performed with the GO Enrichment tool of the BMKCloud platform (www.biocloud.net (accessed on 8 August 2022)). The rhythmicity of genes was analyzed with MetaCycle-meta2d (https://www.rdocumentation.org/packages/MetaCycle/versions/1.2.0/topics/meta2d (accessed on 27 May 2021)) based on the R program [29]. Genes were considered rhythmic if the *p*-value < 0.05. Gene set enrichment analysis (GSEA) is performed by clusterProfiler in R program [30]. Gene set enrichment scores were considered as up-regulation or down-regulation if nominal *p* < 0.05 and cut-off false discovery rate < 0.5. Two-Way ANOVA was conducted to determine the significance of the differentially expressed levels of the genes between CTL and TLE mice with a MATLAB program ‘anova2’, and a *t*-test was conducted to compare the differential expression of genes at ZT7 between CTL and TLE mice with GraphPad (https://www.graphpad.com/quickcalcs/ttest1/ (accessed on 9 June 2021)). The phases, amplitudes, and periods of rhythmicity-maintaining genes were calculated by BioDare2 (https://biodare2.ed.ac.uk/ (accessed on 13 March 2021)) [31]. ‘Liner detrending’ was selected for input data treatment, and the ‘MFourFit’ method was used in the period analysis. Names of genes were converted to EnsemblID using the gProfiler platform (https://biit.cs.ut.ee/gprofiler/convert (accessed on 26 May 2021)) [32]. The 5′ 5000-bp putative promoter sequences of 64 rhythmically expressed mouse epilepsy genes were batch downloaded from the database (GRCm39) and dataset (mouse) with the BioMart tool in Ensemble (https://www.ensembl.org/index.html (accessed on 12 October 2022)). The putative E-box, D-box, and RORE motifs in these promoter sequences were determined with FIMO (Find Individual Motif Occurrences) under the MEME Suite (https://meme-suite.org/meme/tools/fimo (accessed on 12 October 2022)) [33] scanned with input motif downloaded from JASPAR (http://jaspar.genereg.net/ (accessed on 12 October 2022)) [34]. Only motifs having a 99.99% match were selected for the analysis.

## Data Availability

The original contributions presented in the study are included in the article/Supplementary Material, further inquiries can be directed to the corresponding author.

## Supplementary Materials

The following supporting information can be downloaded at: https://www.mdpi.com/article/10.3390/ijms24076400/s1.

## References

[1] Engel J. (2001). A proposed diagnostic scheme for people with epileptic seizures and with epilepsy: Report of the ILAE Task Force on Classification and Terminology. Epilepsia.

[2] Mirzoev A., Bercovici E., Stewart L.S., Cortez M.A., Snead O.C., Desrocher M. (2012). Circadian profiles of focal epileptic seizures: A need for reappraisal. Seizure.

[3] Dębski K., Ceglia N., Ghestem A., Ivanov A., Brancati G., Bröer S., Bot A., Müller J., Becker A., Löscher W. (2020). The circadian dynamics of the hippocampal transcriptome and proteome is altered in experimental temporal lobe epilepsy. Sci. Adv..

[4] Hofstra W.A., Spetgens W.P., Leijten F.S., van Rijen P.C., Gosselaar P., van der Palen J., de Weerd A.W. (2009). Diurnal rhythms in seizures detected by intracranial electrocorticographic monitoring: An observational study. Epilepsy Behav..

[5] Zhang T., Yu F., Xu H., Chen M., Chen X., Guo L., Zhou C., Xu Y., Wang F., Yu J. (2021). Dysregulation of REV-ERBα impairs GABAergic function and promotes epileptic seizures in preclinical models. Nat. Commun..

[6] Li P., Fu X., Smith N.A., Ziobro J., Curiel J., Tenga M.J., Martin B., Freedman S., Cea-Del Rio C.A., Oboti L. (2017). Loss of CLOCK Results in Dysfunction of Brain Circuits Underlying Focal Epilepsy. Neuron.

[7] Sun S., Wang H. (2023). Clocking Epilepsies: A Chronomodulated Strategy-Based Therapy for Rhythmic Seizures. Int. J. Mol. Sci..

[8] Zeng H., Jia J., Liu A. (2010). Coordinated translocation of mammalian Gli proteins and suppressor of fused to the primary cilium. PLoS ONE.

[9] Masson J., Emerit M.B., Hamon M., Darmon M. (2012). Serotonergic signaling: Multiple effectors and pleiotropic effects. Wiley Interdiscip. Rev. Membr. Transp. Signal..

[10] Brandon N.J., Jovanovic J.N., Colledge M., Kittler J.T., Brandon J.M., Scott J.D., Moss S.J. (2003). A-kinase anchoring protein 79/150 facilitates the phosphorylation of GABAA receptors by cAMP-dependent protein kinase via selective interaction with receptor β subunits. Mol. Cell. Neurosci..

[11] Chen C., Yang J.M., Hu T.T., Xu T.J., Xu W.P., Wei W. (2013). Elevated dopamine D2 receptor in prefrontal cortex of CUMS rats is associated with downregulated cAMP-independent signaling pathway. Can. J. Physiol. Pharmacol..

[12] Jijón-Lorenzo R., Caballero-Florán I.H., Recillas-Morales S., Cortés H., Avalos-Fuentes J.A., Paz-Bermúdez F.J., Erlij D., Florán B. (2018). Presynaptic Dopamine D2 Receptors Modulate [(3)H]GABA Release at StriatoPallidal Terminals via Activation of PLC → IP3 → Calcineurin and Inhibition of AC → cAMP → PKA Signaling Cascades. Neuroscience.

[13] Carlson A.B., Kraus G.P. (2022). Physiology, Cholinergic Receptors. StatPearls.

[14] Bläsche R., Ebeling G., Perike S., Weinhold K., Kasper M., Barth K. (2012). Activation of P2X7R and downstream effects in bleomycin treated lung epithelial cells. Int. J. Biochem. Cell Biol..

[15] Ayoub M.A., Angelicheva D., Vile D., Chandler D., Morar B., Cavanaugh J.A., Visscher P.M., Jablensky A., Pfleger K.D., Kalaydjieva L. (2012). Deleterious GRM1 mutations in schizophrenia. PLoS ONE.

[16] Chu H., Sun P., Yin J., Liu G., Wang Y., Zhao P., Zhu Y., Yang X., Zheng T., Zhou X. (2017). Integrated network analysis reveals potentially novel molecular mechanisms and therapeutic targets of refractory epilepsies. PLoS ONE.

[17] Frank M., Kemler R. (2002). Protocadherins. Curr. Opin. Cell Biol..

[18] Dibbens L.M., Tarpey P.S., Hynes K., Bayly M.A., Scheffer I.E., Smith R., Bomar J., Sutton E., Vandeleur L., Shoubridge C. (2008). X-linked protocadherin 19 mutations cause female-limited epilepsy and cognitive impairment. Nat. Genet..

[19] Hoshina N., Johnson-Venkatesh E.M., Hoshina M., Umemori H. (2021). Female-specific synaptic dysfunction and cognitive impairment in a mouse model of PCDH19 disorder. Science.

[20] Mattison K.A., Tossing G., Mulroe F., Simmons C., Butler K.M., Schreiber A., Alsadah A., Neilson D.E., Naess K., Wedell A. (2022). ATP6V0C variants impair vacuolar V-ATPase causing a neurodevelopmental disorder often associated with epilepsy. Brain A J. Neurol..

[21] Anttila V., Bulik-Sullivan B., Finucane H.K., Walters R.K., Bras J., Duncan L., Escott-Price V., Falcone G.J., Gormley P., Malik R. (2018). Analysis of shared heritability in common disorders of the brain. Science.

[22] Bonsall D.R., Lall G.S. (2013). Protein kinase C differentially regulates entrainment of the mammalian circadian clock. Chronobiol. Int..

[23] Lemmer B., Barmeier H., Schmidt S., Lang P.H. (1987). On the daily variation in the beta-receptor-adenylate cyclase-cAMP-phosphodiesterase system in rat forebrain. Chronobiol. Int..

[24] Osonoe K., Ogata S., Iwata Y., Mori N. (1994). Kindled amygdaloid seizures in rats cause immediate and transient increase in protein kinase C activity followed by transient suppression of the activity. Epilepsia.

[25] Ping X., Qin S.K., Liu S.N., Lu Y., Zhao Y.N., Cao Y.F., Zhang Y.H., Zhang S.D., Chu L., Pei L. (2019). Effects of Huazhuo Jiedu Shugan Decoction on Cognitive and Emotional Disorders in a Rat Model of Epilepsy: Possible Involvement of AC-cAMP-CREB Signaling and NPY Expression. Evid.-Based Complement. Altern. Med. Ecam.

[26] Arshad M.N., Naegele J.R. (2020). Induction of Temporal Lobe Epilepsy in Mice with Pilocarpine. Bio-protocol.

[27] Hughes M.E., Abruzzi K.C., Allada R., Anafi R., Arpat A.B., Asher G., Baldi P., de Bekker C., Bell-Pedersen D., Blau J. (2017). Guidelines for Genome-Scale Analysis of Biological Rhythms. J. Biol. Rhythm..

[28] Bu D., Luo H., Huo P., Wang Z., Zhang S., He Z., Wu Y., Zhao L., Liu J., Guo J. (2021). KOBAS-i: Intelligent prioritization and exploratory visualization of biological functions for gene enrichment analysis. Nucleic Acids Res..

[29] Wu G., Anafi R.C., Hughes M.E., Kornacker K., Hogenesch J.B. (2016). MetaCycle: An integrated R package to evaluate periodicity in large scale data. Bioinformatics.

[30] Wu T., Hu E., Xu S., Chen M., Guo P., Dai Z., Feng T., Zhou L., Tang W., Zhan L. (2021). clusterProfiler 4.0: A universal enrichment tool for interpreting omics data. Innovation.

[31] Zielinski T., Moore A.M., Troup E., Halliday K.J., Millar A.J. (2014). Strengths and limitations of period estimation methods for circadian data. PLoS ONE.

[32] Raudvere U., Kolberg L., Kuzmin I., Arak T., Adler P., Peterson H., Vilo J. (2019). g:Profiler: A web server for functional enrichment analysis and conversions of gene lists (2019 update). Nucleic Acids Res..

[33] Grant C.E., Bailey T.L., Noble W.S. (2011). FIMO: Scanning for occurrences of a given motif. Bioinformatics.

[34] Castro-Mondragon J.A., Riudavets-Puig R., Rauluseviciute I., Lemma R.B., Turchi L., Blanc-Mathieu R., Lucas J., Boddie P., Khan A., Manosalva Pérez N. (2022). JASPAR 2022: The 9th release of the open-access database of transcription factor binding profiles. Nucleic Acids Res..

