# Supplementary material for: Reprogramming the Circadian Dynamics of Epileptic Genes in Mouse Temporal Lobe Epilepsy
Source: Int J Mol Sci. 2023 Mar 29;24(7):6400. doi: 10.3390/ijms24076400 (PMC10094901; doi:10.3390/ijms24076400)
Supplement: Supplementary file 1 [file ijms-24-06400-s001.zip › Supplementary FIgure&Tables.pdf]

International Journal of *Molecular Sciences*

MDPI

Supplementary figure and tables

Reprogramming the circadian dynamics of epileptic genes in mouse temporal lobe epilepsy

Sha Sun [1,2] and Han Wang [1,2,*]

[1] Center for Circadian Clocks, Soochow University, Suzhou 215123, China; sunsha@suda.edu.cn
[2] School of Biology & Basic Medical Sciences, Suzhou Medical College, Soochow University, Suzhou 215123, China
* Correspondence: han.wang88@gmail.com or wanghan@suda.edu.cn; Tel.: +86-186-0512-8971

Table S1. Classification of 251 arrhythmic mouse epilepsy-related genes into 60 driver genes, 71 passenger genes, and 120 undetermined genes.

	**Arrhythmicity**
Driver genes	*Scn1a, Scn2a1, Chrna4, Cdkl5, Efhc1, Cacna1h, Chrna2, Polg, Nrxn1, Cntnap2, Epm2a, Arx, Grik2, Mef2c, Kcna2, Kcnq3, Aldh7a1, Gria2, Grin2a, Foxg1, Gabrb1, Cstb, Chrna7, Slc9a6, Kcnab1, Piga, Zeb2, Smc1a, Rest, Slc6a1, Cacnb4, Nr2f1, Trpm2, Tpp1, Cln8, Gli3, Kcnmb3, Myh1, Alg14, Kcnt2, Arv1, Ugp2, Casr, Guf1, Prdm8, Ywhag, Asah1, Necap1, Pigq, Slc13a5, Neurod2, Gosr2, Lmnb2, Kcnb1, Pigp, Fbxo28, Ick, Grin1, Celf2, Hcn4*
Passenger genes	*Il1b, Bdnf, Kcnj10, Idh1, Tnf, Vdr, Cacna1g, Slc6a11, Stx1a, Gstm2, Ankk1, Maoa, Il1rn, Kcnj3, Ache, Myh6, Iqsec2, Vrk2, Zdhhc9, Scn2b, Kcnt1, Nipa2, Cd40, Gab2, Tnfsf10, Gpr98, Casp8, Cul4b, Ap1s2, Rora, Reln, Scn4b, Scn5a, Scn7a, Upf3b, Mboat7, Ppdpf, Ube2a, C3, Trim3, Chrm3, Chrna5, Clcn1, Ddhd2, Akt1, Gabra3, Emp1, Drd4, Glul, Fas, Htr2a, Nfe2l2, Nnmt, Aqp4, Inha, Cdk19, Cpa6, Grin2b, Pigb, Wwox, Itpa, Ager, Got2, Prl, Hif1a, Ins2, Rasal2, Bai3, Adora2a, Nlrp1b, Flna*
Undetermined genes	*Abcb1a, Cyp2c65, H2-K1,H2-Bl,H2-Q4, H2-Q6, H2-Q7, H2-Q1, H2-Q2, Ephx1, Abcg2, Cyp3a11, Cyp3a44, Scn3a, Il1a, Clcn2, Ugt1a1, Cyp1a2, Cyp2c39, Htr1a, Kcnj11, H2-Ab1, Htr1b, Dbh, Gabrd, Cyp2b10, Slco2b1, Abcc4, Ralbp1, Tgfb1, Slc16a1, Pparg, Rorb, Ptger3, Scn3b, Cacna1i, Cat, Slc16a7, Cd36, Slc25a27, Keap1, Slc7a3, Kat2b, Sh3bgrl2, Efhc2, Per2, Allc, Ptgs2, Bad, Rplp2, Spg21, Pmm2, Nde1, Prkcd, Nadsyn1, Abcb4, Slc1a6, Ccl12, Tor3a, Shmt1, Slc22a4, Slc2a3, Sp1, Fa2h, Usf1, Timp4, Tcn2, Trpc4, Cetn3, Mbd6, Clcn7, Cyp2r1, Clcn5, Glyctk, Unc5d, Mblac2, Cyp27a1, Cyp27b1, Akr1b3, Ddhd2, Cln8, F5, Hepacam, Gsta2, Fdps, Fgfr1, Agtr1a, Dcx, Nags, Gss, Gsta3, Pde7b, Srpx2, Gba, Gnl3, App, Cdkl4, Htr2c, Gstm4, H2-Aa, Cfh, Ddr2, Nmbr, Myh11, Mmp9, Kcnj9, Ido1, Irs1, Abcc6, Gstk1, Kcna1, Rhoa, Cyp4f13, Ugdh, Kcnk4, Gal, Atn1, Mthfs, Nacc1, Esr1, Fam19a2, Col1a1*

Table S2. Classification of 64 rhythmic mouse epilepsy-related genes into 19 driver genes, 29 passenger genes, and 16 undetermined genes.

	**Driver genes**	**Passenger genes**	**Undetermined genes**
Loss-of-rhythmicity genes in mouse TLE	*Kcnq2, Pcdh19, Slc2a1, Aldh2, Trappc6b*	*Vamp2, Idh2, Pcdh7, Chrna3, Park2, Notch4，Drd2, Acap3, Nedd9, Tgfbr1*	*H2-Q10, Slc6a4, Scn9a, Grm4, Cbs, Tert, Cyp1b1, Sv2c, Htr1d, Ophn1*
Gain-of-rhythmicity genes in mouse TLE	*Hcn2, Lgi4, Tbc1d24, Atp6v0c, Mfsd8, Abat, P2rx7, Notch3, Atp1a2, Lepr, Setd1a, Slc38a3, Pigs*	*Abcc1, Tap1, Il6ra, Mvp, Irs2, Slc6a3, Dhcr7, Gsr, Grm1, Gc, Mthfd1, Itgb3, Kcnq1, Pars2, Cux2, Nlrp3*	*Gpx1, Per3, Slc26a4, Atp7b, Mtrr*
Rhythmicity-maintaining genes in mouse TLE	*Samd12*	*Chd2, Abcb6, Rnf13*	*Apeh*

Table S3. Rhythmicity analysis of the ventral hippocampal transcriptome of TLE and CTL mice

Table S4. Two-Way ANOVA of differentially expressed genes between CTL and TLE mice and T-test analysis of them at ZT7

Table S5. GSEA of 315 epilepsy-related genes at ZT7

Table S6. Top 20 KEGG pathways of driver genes

Table S7. Top 20 KEGG pathways of arrhythmicity genes

Table S8. Top 20 KEGG pathways of loss-of-rhythmicity genes

Table S9. Top 20 GO terms of genes of loss-of-rhythmicity genes

Table S10. Top 20 KEGG pathways of gain-of-rhythmicity genes

Table S11. Top 20 GO terms of gain-of-rhythmicity genes

Table S12. Phases, amplitudes, and periods of rhythmicity-maintaining genes

Table S13. The circadian clock-regulated motifs in these 64 rhythmically expressed epilepsy genes

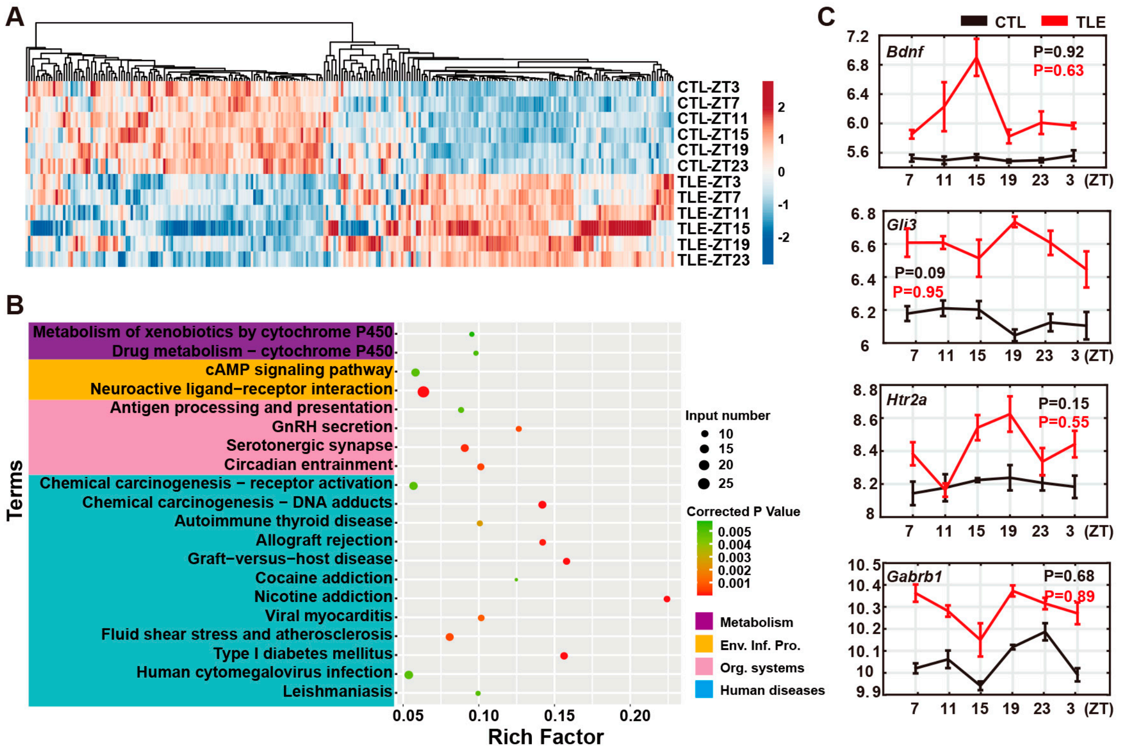

Figure S1. Analysis of arrhythmic mouse epilepsy-related genes. (A) Heatmap of 251 arrhythmicity genes in the ventral hippocampus of TLE and wild-type CTL mice. Down-regulation (blue) and up-regulation (red) are indicated. (B) Top 20 KEGG enrichment pathways of significantly differentially expressed genes in the arrhythmicity group. (C) Expression of representative arrhythmic genes involved in the cAMP signaling pathway, including *Bdnf* (Brain-derived neurotrophic factor) and *Gli3* (GLI-Kruppel family member GLI3), and the serotonergic synapse pathway, including *Htr2a* (5-hydroxytryptamine receptor 2A) and *Gabrb1* (GABA receptor A subunit β1). The black curve represents gene expression values from the CTL mice, and the red curve is for those in TLE mice.
